# First Long-Term Behavioral Records from Cuvier’s Beaked Whales (*Ziphius cavirostris*) Reveal Record-Breaking Dives

**DOI:** 10.1371/journal.pone.0092633

**Published:** 2014-03-26

**Authors:** Gregory S. Schorr, Erin A. Falcone, David J. Moretti, Russel D. Andrews

**Affiliations:** 1 Cascadia Research Collective, Olympia, Washington, United States of America; 2 Naval Undersea Warfare Center, Newport, Rhode Island, United States of America; 3 School of Fisheries and Ocean Sciences, University of Alaska Fairbanks, Fairbanks, Alaska, United States of America; 4 Alaska SeaLife Center, Seward, Alaska, United States of America; Texas A&M University-Corpus Christi, United States of America

## Abstract

Cuvier’s beaked whales (*Ziphius cavirostris*) are known as extreme divers, though behavioral data from this difficult-to-study species have been limited. They are also the species most often stranded in association with Mid-Frequency Active (MFA) sonar use, a relationship that remains poorly understood. We used satellite-linked tags to record the diving behavior and locations of eight *Ziphius* off the Southern California coast for periods up to three months. The effort resulted in 3732 hr of dive data with associated regional movements – the first dataset of its kind for any beaked whale – and included dives to 2992 m depth and lasting 137.5 min, both new mammalian dive records. Deep dives had a group mean depth of 1401 m (s.d. = 137.8, n = 1142) and duration of 67.4 min (s.d. = 6.9). The group mean time between deep dives was 102.3 min (s.d. = 30.8, n = 783). While the previously described stereotypic pattern of deep and shallow dives was apparent, there was considerable inter- and intra-individual variability in most parameters. There was significant diel behavioral variation, including increased time near the surface and decreased shallow diving at night. However, maximum depth and the proportion of time spent on deep dives (presumed foraging), varied little from day to night. Surprisingly, tagged whales were present within an MFA sonar training range for 38% of days locations were received, and though comprehensive records of sonar use during tag deployments were not available, we discuss the effects frequent acoustic disturbance may have had on the observed behaviors. These data better characterize the true behavioral range of this species, and suggest caution should be exercised when drawing conclusions about behavior using short-term datasets.

## Introduction

The beaked whales (family *Ziphiidae*), Cuvier’s beaked whale (*Ziphius cavirostris,* hereafter *Ziphius)* in particular, have exceptional diving capabilities. Though it is the most broadly distributed beaked whale species, they remain poorly understood, as their behavior, especially their preference for deep water habitat typically far from shore, makes them notoriously difficult to study. To date, published dive data from *Ziphius* have come primarily from short-term data logging tags deployed in three different regions: the Ligurian Sea, Hawaii, and most recently Southern California. These deployments have averaged only 12 hr in length and in total represent less than 215 hrs and 327 dives [1]–[3]. Despite their limitations, these data suggest *Ziphius* routinely conduct some of the deepest and longest dives of any mammal [1], [2], many of which exceed the estimated aerobic dive limit for the species by a factor of two or more [2]. *Ziphius* also appear unusually sensitive to acoustic disturbance; it is the species most frequently stranded coincident with Mid-Frequency Active (MFA) sonar exposure during military exercises [4]. Though it has generated a great deal of scientific interest, the causal relationship between these strandings and sonar remains elusive [3], [5].

**Figure 1:**
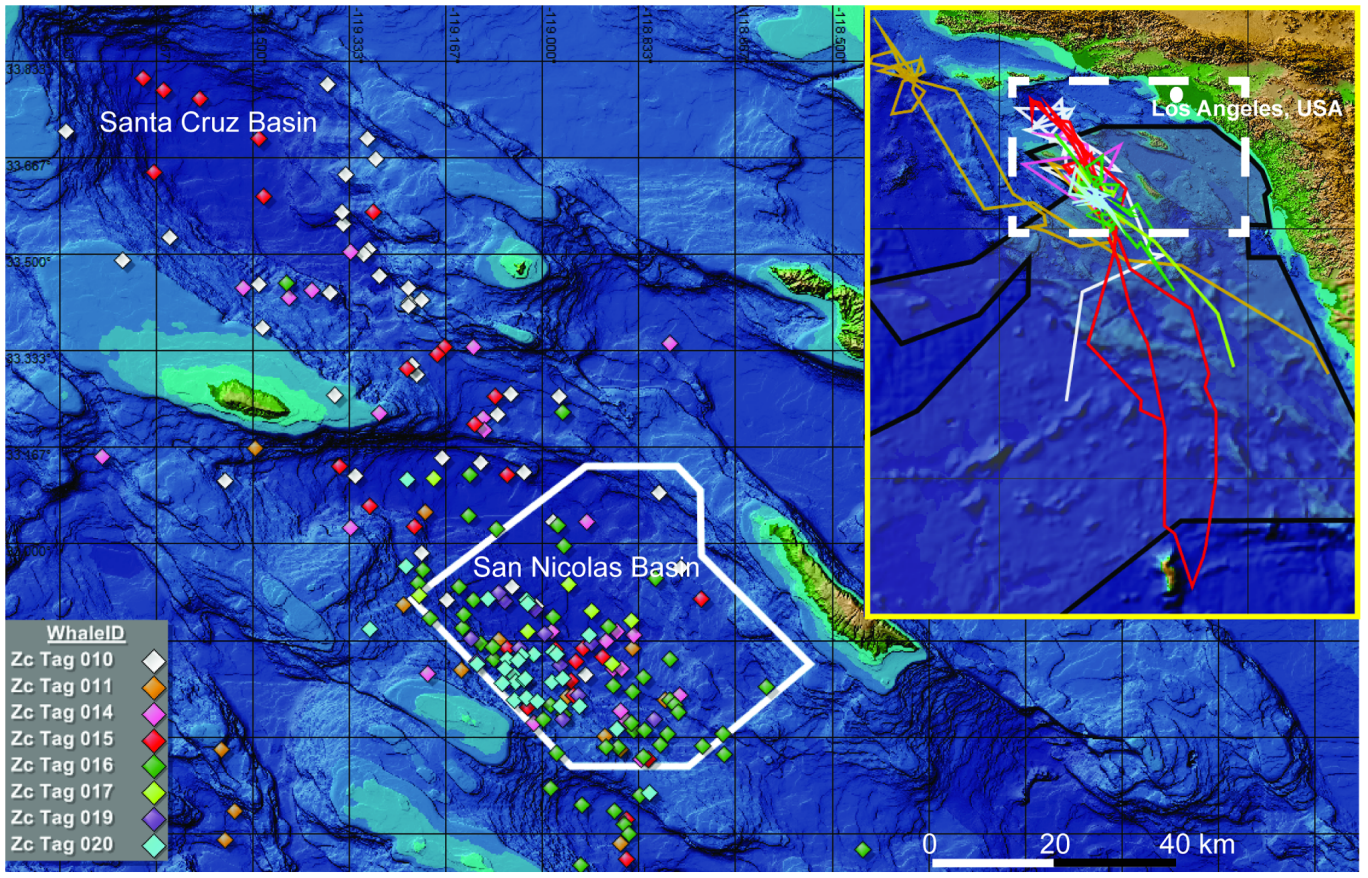
Map of the study area displaying one daily position estimate of each tagged whale. All eight whales were tagged within the Southern California Anti-submarine Warfare Range (outlined in solid white). Tagged whales were within the San Nicolas Basin for 51% of all days tags transmitted, and within the SOAR boundaries for 71% of the days when in the basin, suggesting site fidelity to the MFA sonar training range. Inset map shows the entire track-line of each tagged whale with the primary map region indicated by a dashed box. The SOCAL Range Complex, which encompasses all areas of authorized MFA sonar use [6], is lightly shaded and outlined in black in the inset map. Map created using Mysticetus Observation System, v1.8.0.124 (Entiat River Technologies, Preston, WA, USA).

The U.S. Navy utilizes the Southern California Range Complex (SOCAL Range Complex) to conduct various training activities [6], including Anti-Submarine Warfare (ASW) training which frequently includes the use of MFA sonar (Figure 1). Between 2009 and 2014, the Navy is authorized to use up to 3408 hours of MFA sonar annually [7]. While MFA use can occur anywhere within the SOCAL Range Complex, the Southern California Anti-submarine Warfare Range (SOAR) (Figure 1), is specifically instrumented to facilitate ASW training, with 172 bottom-mounted hydrophones covering nearly 1800 km^2^.

A recent study has sought to directly measure the behavioral responses of *Ziphius* to MFA by exposing suction-cup tagged whales to a simulated naval sonar signal. The first two successful playback experiments of this type occurred within the SOCAL Range Complex, and in both experiments, the tagged whale responded strongly to simulated sonar at received levels as low as 89 dB re 1 μPa [3]. However, one whale was also incidentally exposed to real naval sonar from a distant ship at a received level of 106 dB re 1 μPa and showed no response, suggesting that the behavior of this species in the presence of sonar is likely influenced by more than simply received level [3]. Ultimately, the implications of the behavioral responses captured in these short-term tagging records are limited by a lack of long-term context in which they occur, since the behavior of individual *Ziphius* beyond a matter of hours post-tagging has not been documented.

In 2006, we began a cooperative visual-acoustic study combining the Marine Mammal Monitoring on Navy Ranges (M3R) program [8] and small boat visual surveys in order to identify which cetacean species utilize the SOAR range. Surprisingly, 37 *Ziphius* groups were encountered on the SOAR range during five surveys between 2006–2008, and re-sightings of photo-identified individuals suggested some degree of site-fidelity to this MFA training range [9]. In an effort to better characterize the distribution and diving behavior of *Ziphius* on the SOAR range, satellite tagging was added to the study as the technology became available. Here we describe the first long-term records of diving behavior from eight *Ziphius* tagged on SOAR (Figure 1), which provide insight into the true behavioral range of this species in a region of regular acoustic disturbance. We calculate basic parameters of dives and intervening surfacing bouts, describe patterns in the dive/surfacing cycle, and assess variability within and between individuals. We compare these values to published data from this and other regions, and discuss the relevance of these data to studies of the behavioral impacts of MFA sonar.

## Materials and Methods

### Ethics Statement

All field work was conducted under U.S. National Marine Fisheries Service research permits No. 540–1811 and 16111. Tagging was approved by the Cascadia Research Collective Institutional Animal Care and Use Committee.

### Fieldwork

Small boat surveys were conducted as described in Falcone *et al.* [9]. We used a modified air rifle to deploy Mk10-A Argos-linked dive recorders (Wildlife Computers, Redmond, WA, USA) in the Low Impact Minimally Percutaneous External-electronics Transmitter (LIMPET) configuration [10], [11] on or near the dorsal fin. The tags were affixed to the dorsal fin by two barbed darts constructed from medical-grade titanium which were gas-sterilized prior to implantation. Tagged whales were assigned to putative sex and age classes based on body size, pigmentation, and markings from photographs taken at the time of deployment, and compared to an existing regional catalog to assess sighting history, age and sex [9].

### Tag Data

Dive data were collected using the Wildlife Computers Behavior Log (BL) function [12], which compiled records of user-defined “dives” and “surfacing bouts” and transmitted them via the Argos satellite system. Gaps in the BL occurred when one or more Argos message failed to be received due to factors such as no satellite coverage, surfacing behavior, weather, or duty cycling. Dives were defined as any submergence that exceeded 50 m depth and lasted longer than 30 s, and each dive record provided the start time, end time, maximum depth reached, and dive shape. Dive shapes were automatically classified by Wildlife Computers’ Dive Analysis Program (DAP) version 3.0 build 141 [13] as “Square”, “V”, or “U” based on the proportion of time spent in the bottom phase of the dive, and were used to create a visual representation of the BL as an approximated dive trace. Maximum dive depths were reported in resolution steps of up to +/−1.5% of the actual maximum depth measured during the dive. The depth accuracy for the type of pressure transducers used in these tags was independently verified in a pressure chamber to 3000 m, which demonstrated a maximum error of<+/−2.5% of the recorded value.

Surfacing bouts represented the time between qualifying dives (i.e., when the whale did not descend below 50 m for more than 30 s), and included the start/end times only. Start/end times were determined by the transition from wet to dry at the surface for five tags, and crossing the 5 m depth threshold upon descent or ascent of a qualifying dive for the remaining three tags (Table 1). Because surfacing bouts were typically brief, the average value of surfacing bout durations differed significantly between tags using the wet/dry sensor and tags using the depth sensor to log start/end times (median 1.83 min wet/dry *n* = 3380, versus median = 2.39 min depth *n* = 3429, Mann-Whitney U Test, *p*<0.001). Thus statistical analyses of surfacing bout duration used only wet/dry sensor tags, as these more accurately characterize short respiratory bouts. The durations of dives, which were much longer than surfacing bouts, did not vary significantly by sensor type.

**Table 1:** Summary of satellite tag deployments by individual.

Whale ID	AgeClass	Sex	Event Start/EndTime By	DeploymentDate	TransmissionDuration (days)	Total BLData (hr)	Filtered ArgosPositions (n)	Distance to Deployment Location(km) median (range)
Zc010	Adult	Female	Wet/Dry	29Jun2010	53.6	499.2	171	68.1 (5.3–265.5)
Zc011	Adult	Male	Wet/Dry	29Jun2010	89.8	544.7	174	198.7 (3.5–289.5)
Zc014	Sub-adult	Unknown	5 m Depth	06Jan2011	22.5	91.2	80	17.5 (2.4–94.4)
Zc015	Adult	Female	5 m Depth	06Jan2011	70.6	1015.9	291	83.8 (3.5–452.3)
Zc016	Adult	Male	5 m Depth	06Jan2011	88.7	857.5	194	20.0 (0.8–103.2)
Zc017	Adult	Female	Wet/Dry	23Jul2011	9.7	110.1	42	29.2 (6.3–235.7)
Zc019	Adult	Female	Wet/Dry	15Jan2012	12.0	172.5	49	12.2 (0.9–33.1)
Zc020	Adult	Male	Wet/Dry	15Jan2012	26.4	441.2	122	16.8 (0.8–49.7)

Tags were programmed to transmit daily for 28 days, and then to transmit on alternating days thereafter (duty cycle) to extend battery life (at the expense of data continuity later in the transmission period). Argos position estimates were filtered for plausibility with the Douglas Argos Filter [14] using the same parameters outlined by Schorr *et al.* [11]. The single best daily position estimates per whale (highest quality Argos location), as determined by the Douglas Argos Filter, were used to generate Figures 1 and 5; all other spatial analyses used the complete set of Argos positions that passed the filter.

BL records were assigned to day (solar elevation >0) or night classes (solar elevation <0) by calculating the solar elevation at the start time and the location of the nearest filtered Argos position estimate in time. Solar elevation was calculated using tools available from http://www.esrl.noaa.gov/gmd/grad/solcalc/calcdetails.html (accessed 21 August 2011). Geographic analyses were conducted using the Mysticetus Observation System, v1.8.0.124 (Entiat River Technologies, Preston, WA, USA).

### Dive and Surfacing Bout Classification

**Figure 2:**
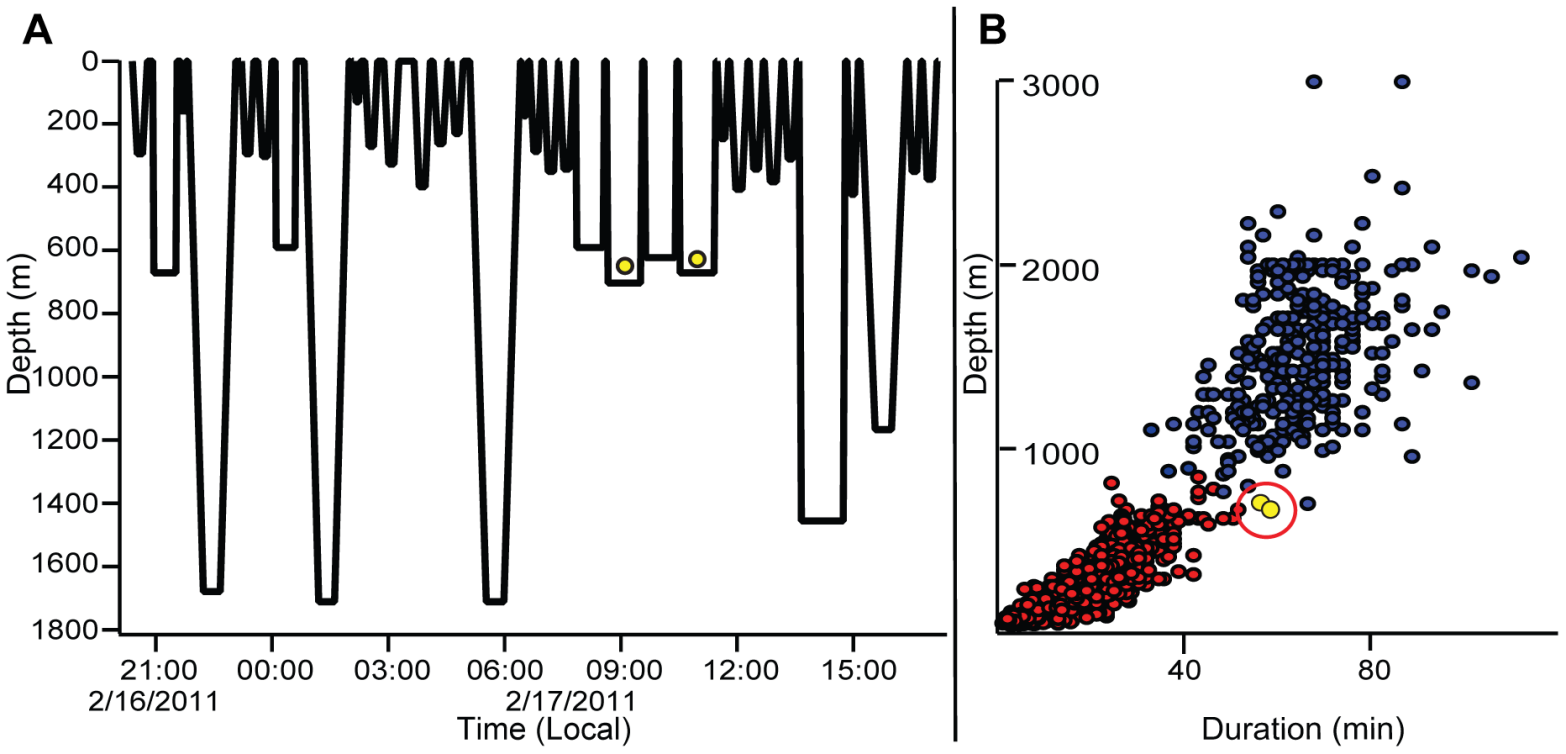
Classification of dive type. (**A**)Sample dive trace from Zc015 containing two reclassified dives. The four consecutive dives in the series beginning at 07∶49 were all unusually long for shallow dives, but unusually shallow for deep dives, and thus difficult to classify using depth and duration alone. The K-means cluster analysis for Zc015 (**B**) classified the two dives indicated by yellow circles in each panel as deep dives and the other two as shallow dives. All four of these dives in figure A were similar and presumably of the same class. Other deep dives in the adjacent period were up to 1000 m deeper than these two dives, and four deep dives in series would be exceedingly rare based on this dataset and prior studies. Thus, we believe all four of these dives were much more likely to be atypically long, shallow dives, and the two indicated with yellow circles were reclassified as such.

Previous authors have partitioned beaked whale dives beyond 50 m into two classes based on their depth, duration, and the presence of foraging behavior as indicated by acoustic and/or fine-scale movement data [1], [2]: long, deep dives associated with foraging by the presence of echolocation clicks and/or evidence of prey chases; and intervening bouts of shorter, shallower, silent dives with no indication of prey searching or chase (the purpose of which remains unclear [15]–[17]). Based on these descriptions, we performed a K-means cluster analysis on the BL dives for each individual, using dive depth and duration to partition all dives into “deep” and “shallow” classes, though the presence or absence of foraging behavior could not be determined for these dives with the sensors available. While these parameters provided clear separation into deep and shallow clusters for most dives (one-way ANOVA by individual, all *p*-values <0.0001), we visually verified the classification accuracy for all long dives which fell below the 5th percentile of depth or duration for that class and all shallow dives that fell above the 5th percentile for depth or duration for that class by individual dataset based on the pattern of surrounding dives in the record (Figure 2). Where there were gaps in the record surrounding a questionable dive or ambiguity in the pattern, the original K-means classification was retained. Eight total dives (0.1% of all dives) from only two individuals were reclassified from their original K-means cluster.

Following Tyack *et al.* [2], the Inter-Deep Dive Interval (IDDI) was calculated as the time between the end of one deep dive and the start time of the next deep dive when there were no data gaps in the intervening period. The overall deep dive rate was calculated by dividing the total number of deep dives by the total hours of BL data for each whale. The depths and durations of deep and shallow dives, surfacing bout durations and IDDIs were not normally distributed, so individual medians with ranges were calculated and the group mean of these values used to characterize events across all individuals.

Surfacing bouts were classified based on their position within the sequence of deep and shallow dives. Surfacing bouts immediately following a deep dive were called first surfacing bouts, those that separated sequential shallow dives were called intermediate surfacing bouts, those that immediately preceded a deep dive were called terminal surfacing bouts, and those that separated back-to-back deep dives were called single surfacing bouts.

## Results

Eight tags were deployed on *Ziphius* at SOAR from 2010 to 2012, providing data for periods of up to three months (Table 1). The resulting dataset consisted of 3732 hrs of dive and surfacing bout records with 1123 concurrent animal location estimates (Table 1, Figure 1). In total, tagged whales performed 1142 deep dives to a group mean depth of 1401 m and duration of 67.4 min, and 5685 shallow dives averaging 275 m and 21.0 min (Table 2). The deepest dive reached 2992 m, and the longest dive lasted 137.5 min.

**Table 2:** Summary of dive and surfacing bout parameters by individual.

	Deep Dives			Shallow Dives			Surfacing bouts	
Whale ID	*n*	*Depth (m)*	*Duration (min)*	*n*	*Depth (m)*	*Duration (min)*	*n*	*Duration (min)*
**Zc010**	187	1328 (880–1924)	59.2 (24.0–137.5)	908	280 (54–784)	17.6 (1.9–40.0)	1089	1.87 (0.02–38.1)
**Zc011**	129	1464 (768–2295)	78.4 (51.7–114.7)	846	280 (52–880)	24.0 (2.0–57.1)	963	1.59 (0.02–71.1)
**Zc014**	32	1520 (656–1872)	58.7 (36.8–82.7)	148	272 (58–592)	19.5 (2.0–41.1)	180	2.13 (0.50–38.1)*
**Zc015**	315	1488 (704–2992)	64.5 (33.1–112.5)	1617	288 (50–848)	21.3 (1.1–58.1)	1931	2.39 (0.30–205.5)*
**Zc016**	263	1360 (784–1840)	72.0 (35.7–110.4)	1054	312 (50–720)	25.6 (2.1–66.7)	1318	2.39 (0.08–123.1)*
**Zc017**	25	1616 (1072–1840)	76.3 (56.0–99.7)	184	256 (54–576)	19.7 (4.4–37.9)	209	1.99 (0.02–79.7)
**Zc019**	53	1264 (912–1808)	64.5 (38.9–91.2)	282	256 (52–1168)	18.7 (0.9–43.2)	334	1.99 (0.02–70.4)
**Zc020**	138	1168 (768–1872)	65.6 (36.8–114.7)	646	256 (58–784)	21.9 (1.0–57.1)	784	1.93 (0.25–84.8)
**Total**	**1142**	**1401 (137.8)**	**67.4 (6.9)**	**5685**	**275 (18.3)**	**21.0 (2.6)**	**6808**	1.87 (0.17)/2.30 (0.2)*

Individual table values are medians, with ranges in parentheses. * indicates tags that logged the start and end of dives and surfacing bouts when the whale passed through 5 m depth on descent/ascent of qualifying dives; others used the wet/dry sensor on the tag for time stamps. The latter likely represent a more accurate measure of actual respiratory periods in surfacing bout metrics. Datasets are summed and group means (s.d.) are calculated across individuals at the bottom of the table.

**Figure 3:**
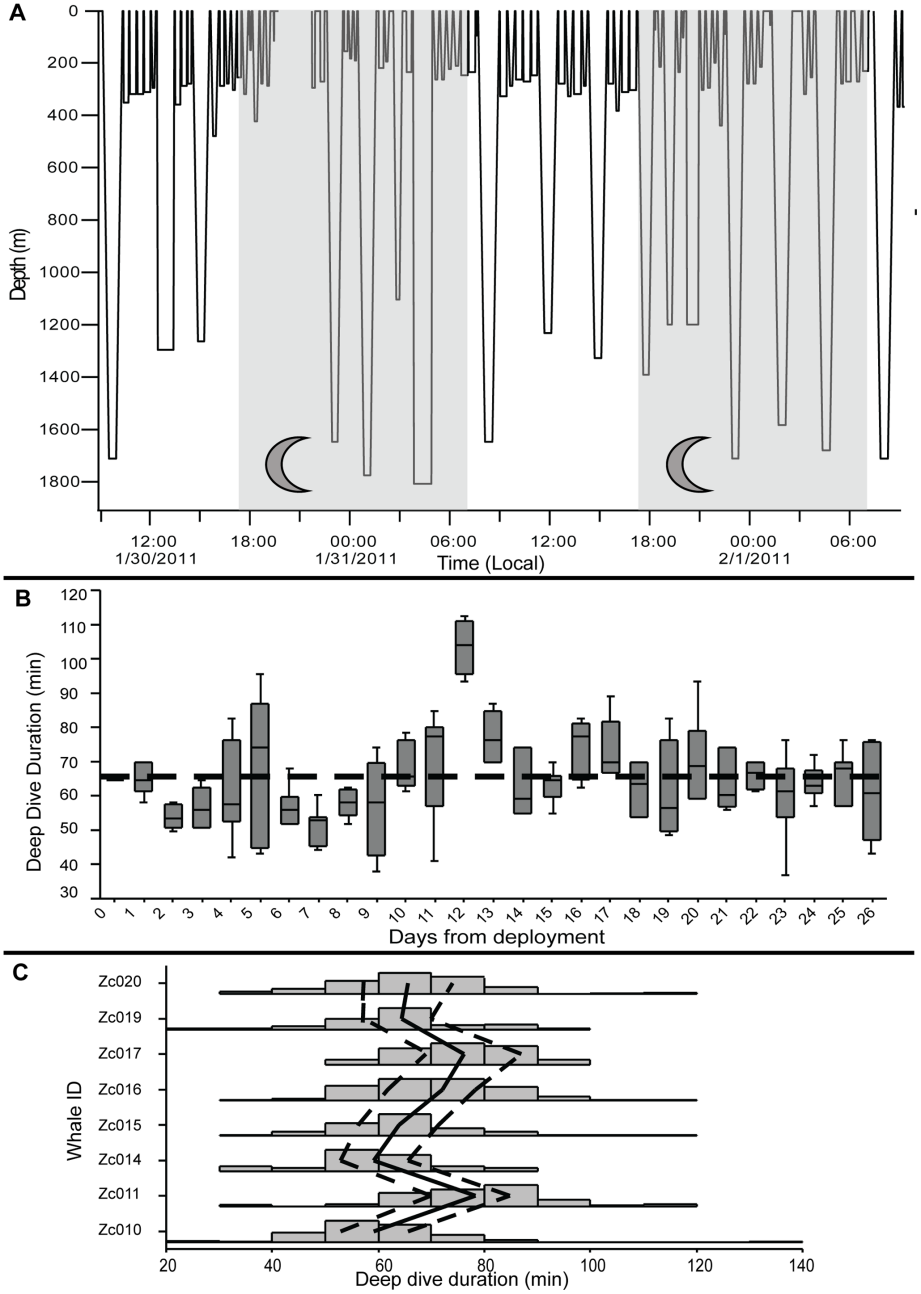
Variation in diving behavior. (**A**) A 48-hr BL dive trace from Zc015, illustrating the pattern of “deep” and “shallow” dives. Darkened areas represent night. The gap in the dive record during the first night period reflects data that were not received via Argos. (**B**) Box plot of deep dive duration by day for the first 26 days of Zc015. The dashed line represents the overall median dive duration for Zc015 of 64.0 min. (**C**) Histogram plot of “deep” dive duration by individual, demonstrating the variability both within and among individuals. The solid black line connects the median value for each individual; the dashed lines connect the upper and lower 25th percentiles.

The stereotypical dive pattern previously described for *Ziphius* – single deep, foraging dives separated by a series of shallow dives [1], [2] (Figure 3A) – was prevalent throughout the dive records of all eight whales, though there was substantial behavioral variation both within and among individuals (Figures 3B,3C). Occasional back-to-back deep dives were performed by all but one whale. In the combined data, deep dives ranged from 656–2992 m in depth and from 24.0–137.5 min in duration (Table 2). The group mean duration of the IDDI was 102 min and consisted of 4.13 shallow dives (Table 3), though this value ranged widely (0–21 shallow dives per IDDI). The group mean rate of deep dives was 0.30 per hour (s.d. = 0.05).

**Table 3:** Inter-deep dive intervals (IDDI) and deep dive rates.

	Inter-Deep Dive-Intervals			
Whale ID	*n*	*Duration (min)*	*# Shallow Dives*	Deep Dives per hr
**Zc010**	127	87.3 (2.7–276.3)	4 (0–10)	0.37
**Zc011**	68	132.2 (2.4–267.1)	5 (0–9)	0.24
**Zc014**	11	33.1 (3.7–116.9)	2 (0–4)	0.35
**Zc015**	249	116.7 (2.8–431.1)	5 (0–15)	0.31
**Zc016**	176	100.0 (26.3–399.2)	4 (0–10)	0.31
**Zc017**	13	139.6 (57.6–227.6)	5 (2–10)	0.23
**Zc019**	34	99.6 (28.6–369.3)	4 (0–21)	0.31
**Zc020**	105	110.4 (25.4–287.5)	4 (0–19)	0.31
**Total**	**783**	**102.3 (30.8)**	**4.13 (0.99)**	**0.3 (0.05)**

Individual table values are medians, with ranges in parentheses. The IDDI represents the period from the end of one deep dive to the beginning of the next deep dive, where there was no gap in dive data. Datasets are summed and group means (s.d.) are calculated across individuals at the bottom of the table.

**Figure 4:**
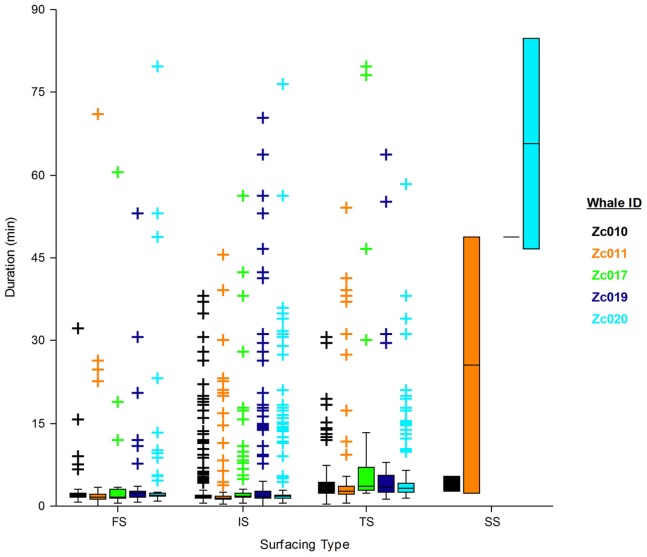
Box plot of surfacing bout duration by surfacing type, by individual. First Surfacing bouts (FS), Intermediate Surfacing bouts (IS), Terminal surfacing bouts (TS), and Single Surfacing bouts between deep dives (SS). Only the five tags using the wet/dry sensor are included (Table 2). Zc019 had only one SS, and Zc017 did not conduct any consecutive deep dives. Boxes represent the median and inter-quartile range, with whiskers at 1.5 times the inter-quartile range. Severe outliers are indicated by plus symbols. Surfacing bouts were typically very short, however occasional severe outliers (representing unusually long surfacing bouts) occurred in all surfacing bout types.

The durations of all surfacing bouts captured by the five tags using the wet/dry sensor were pooled and compared by type (Figure 4). Terminal surfacing bouts were significantly longer (median = 3.13 min, range = 0.33–79.75 *n* = 499) than both first surfacing bouts (median = 1.93 min, range = 0.02–79.73, *n* = 509) and IS (median = 1.66 min, range = 0.02–76.53, *n* = 2188) (Kruskal-Wallis multiple comparison, Z-value >14.6, *α = 0.05*). Terminal surfacing bouts were not significantly longer than single surfacing bouts (Kruskal-Wallis multiple comparison, Z-value = 1.14), but this is likely influenced by small sample size (*n* = 8) and high variability of single surfacing bout durations, which ranged from 2.4–84.8 min in length.

Diel difference in deep dive depth and duration were compared using a paired T-test of the individual median values. Foraging dive depths were significantly deeper at night (1487 m) than during the day (1345 m) (*p* one-tailed = 0.02, *df* = 7, T-value = −2.48), while deep dive durations were longer during the day (69.8 min) than at night (64.9 min) (*p* one-tailed = 0.001, *df* = 7, T-value = 4.53). A series of paired T-tests was run comparing the proportion of time spent in each behavior mode (deep dive, shallow dive and surfacing bout) for each whale during the day and night (Table 4). All tests were significant at *p* = 0.05, some strongly so.

**Table 4:** Diel differences in diving behavior.

Variable	Day	Night	*T*	*p*
Deep Dives per Hour	0.27	0.33	−3.28	<0.01
% Time Surface bout	0.070	0.175	−6.68	<0.01
% Time Shallow Diving	0.610	0.465	8.03	<0.01
% Time Deep Diving	0.320	0.360	−2.28	0.06

Behavior modes were partitioned into day and night classes for each tagged whale, and the total time spent within each class was calculated for each whale by day and night. A series of paired T-tests was then run (*df* = 7 for all tests) to assess diel differences. In general, whales spent more time at the surface at night and more time on shallow dives during the day. Though they conducted significantly more deep dives at night, the total time spent deep diving was not significantly different, as night time deep dives were often shorter in duration.

**Figure 5:**
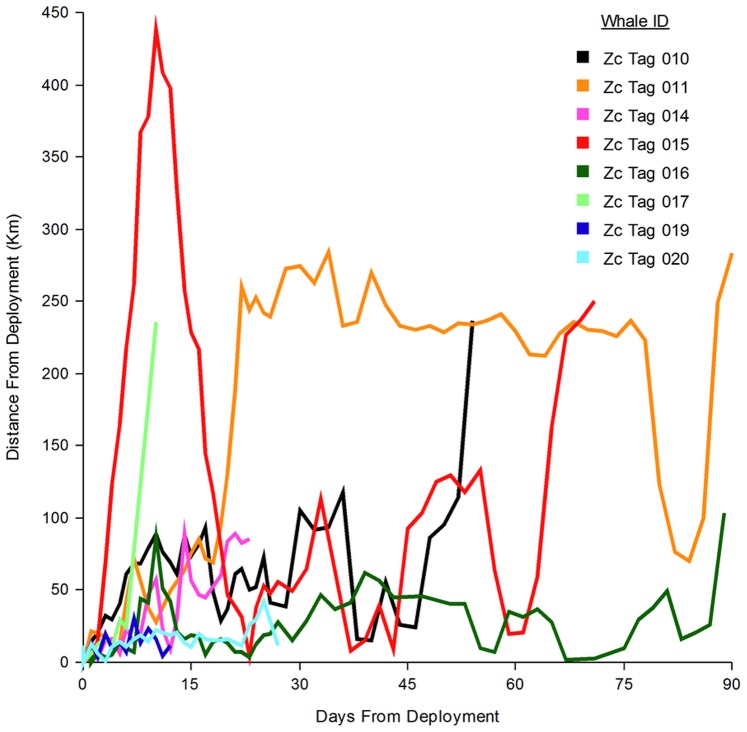
Distance to deployment location from day of deployment, by individual. Distance from deployment was calculated using the best location per day, as determined by the Douglas Argos Filter [14]. All individuals were tagged on the SOAR MFA sonar training range, and most remained in close proximity to the area in which they were tagged. While Zc015 made an extra-regional excursion over 450 km away, she returned to within 5 km of her tagging location.

Tagged whales utilized the San Nicolas Basin, which encompasses SOAR, on over 51% of days location estimates were received (Figure 1) and were documented in the basin during eight different months of the year. Thirty-six percent of all 1123 locations received were within SOAR boundaries. Group mean distance to tag deployment location was 56 km (range = 12–199, *n* = 8) (Table 1, Figure 5).

## Discussion

As with past studies, the animals tagged here exhibited profound diving capabilities; however, the dive depths and durations reported here far exceed the prior records for this species. The deepest dive of 2992 m exceeded the previous maximum depth from this species by 1104 m [2], and the longest dive lasted 137.5 min, a 45% increase over the previous maximum dive duration [18]. These values also represent new mammalian dive depth and duration records, previously 2388 m [19] and 120 min [20] from southern elephant seals.

Surfacing bouts averaged less than 2 min (Table 2), reflecting this species’ exceptionally short gas exchange interval, especially relative to their typically long dives. Surfacing bouts preceding deep dives were significantly, but not dramatically, longer than average (group mean = 3.29 min, *n* = 5), suggesting *Ziphius* may prepare for these longer submergences. Surfacing bouts that separated back-to-back deep dives were among the longest observed as a class (group mean = 35.7 min, range = 2.39–115.7, *n* = 5), though some of these were no longer than average (Figure 4). Extended surfacing bouts, some lasting several hours (max 205.5 min from a depth sensor tag), were uncommon, and occurred at various points in the dive cycle.

Diel patterns in dive behavior were strongly evident in this dataset, confirming previous observations from short overnight deployments [18] that *Ziphius* spend significantly more time in waters above 50 m at night than they do during the day. In fact, all but one of 22 surfacing bouts longer than 60 min occurred at night. Tagged whales spent significantly more time engaged in shallow diving during the day, but significantly less time above 50 m. Though deep dives were significantly deeper at night, the difference was only 142 m. The percentage of time spent deep diving was not significantly different from day to night (Table 4), suggesting whales foraged around the clock, targeting prey that did not vertically migrate.

The overall rate of deep dives (0.30 per hr, s.d. = 0.05), indicated our whales foraged approximately 7 times per day. *Ziphius* in Hawai‘i conducted approximately 10 deep dives daily [1], [18], and whales in the Ligurian Sea conducted 11–12 foraging dives per day [2]. Assuming the deep dive rate approximates foraging effort, whales in this study appear to forage less often – potentially by as much as 40% – than whales in the other study areas. This could be driven by several factors. The deeper and longer dives documented in this study may require longer recovery periods between them, which would in turn reduce the number of deep dives whales can conduct each day. However, on average, deep dives in this study were not dramatically deeper and were of similar duration to those observed elsewhere [18], so this seems unlikely to result in such a comparatively large decrease in foraging effort. Another possibility is that whales in our study area are more successful at foraging than whales elsewhere, and don’t need to forage as often. This could be due to alternate foraging tactics, higher prey densities, or better prey quality. Unfortunately, direct measures of foraging activity are not available from these tags, and even general measures of prey type and density are difficult to collect with existing technology at the depths these whales are diving, so the role these factors play in foraging rate remains unknown, though undoubtedly important.

It is also possible that these apparent differences in regional foraging rates are an artifact of small sample sizes inherent in suction cup tag deployments. This view into longer-term behavioral patterns of *Ziphius* suggests caution should be exercised when comparing short-term behavior records from one tagged individual against others. Most parameters displayed greater variability than limited datasets from this species have captured. Figure 3A is a sample dive trace demonstrating variation in the depth, duration, and frequency of dives in one whale over a relatively short period of 48 hours – which is still a longer continuous period of data than any previous tag deployment on this species has captured [1]–[3]; the reduction in shallow diving and increased surfacing bout durations that occur more commonly at night are also evident. Figure 3B displays variation in deep dive duration within and between days for a single individual over four weeks. The total distributions of deep dive duration also varied considerably among individuals (figure 3C). All of this variation suggests that the behavioral range of this species is broader than their stereotypical patterning might suggest, both within and among individuals, and that a longer-term behavioral record from a given individual is the most appropriate context in which to evaluate a given behavior. Lacking this, an extensive behavioral sample from the population of interest might be the next most suitable alternative to evaluating the magnitude of a behavioral change.

The two *Ziphius* off Southern California that were exposed to simulated MFA sonar in an experimental context both appeared to exhibit strong responses at low received levels when comparing exposure dives to a small number of baseline dives [3]. Responses included increased dive duration, prolonged cessation of foraging, and rapid swimming away from the stimulus, though one whale did not respond similarly to very distant real MFA sonar at similar received levels (due to the disparity in transmission levels between the simulated source and actual Navy sonar). DeRuiter *et al*. [3] suggest that proximity to the source may have accounted for the lack of response to real MFA sonar. If similar behaviors occur in response to real MFA sonar but at closer proximity and/or higher received levels, it is possible that sonar exposure may have contributed to the longer average dive durations and apparently, reduced foraging rates observed in this study. While tags utilized in DeRuiter *et al*. [3] collect data on a very different scale than ours, a comparison of the basic parameters of the reactive behaviors against our more extensive regional data set is warranted, as is an evaluation of how such reactions might influence trends in our data, given the prevalence of sonar in the region.

Seven of the whales tagged in this study conducted a total of 75 dives longer than the longest reactive dive in the experimental exposures [3]; in fact, the longest reactive dive was only about 10 min longer than the median deep dive duration for two of our tagged whales. Thus, the reactive dive durations observed in the experimental exposures do not appear far outside the normal behavioral range of some *Ziphius* in this region. The longest average deep dive duration in our study was associated with the whale (Zc011) that remained farthest from SOAR and spent the most time well outside the area where the U.S. Navy is authorized to use MFA (Figures 1 and 5, Tables 1 and 2) [6]. These observations suggest that MFA exposure is unlikely to be a primary factor in the long average dive durations from this dataset, though it may influence some dives.

The post-exposure IDDIs in the experimentally-exposed whales (6.6 and 7.6 hrs) [3] were, however, on par with the longest IDDI captured in this dataset (7.2 hrs) (Table 3), and thus did likely represent a major deviation from normal behavioral patterning for whales in this region. If sonar exposure is responsible for the longer average IDDI in our data, it would negatively influence foraging rates. As whales in this region may be conducting upwards of 40% fewer foraging dives than *Ziphius* elsewhere [2], [18], the impact of sonar could be significant if foraging success and caloric needs are otherwise similar among these populations. However, if we again look to Zc011 as an example, its foraging rate was among the lowest of all tagged whales (0.24 deep dives per hr), and it was arguably the least likely to have been exposed to MFA sonar during most of the deployment (Figure 1), suggesting that factors other than sonar also influence IDDI.

All whales tagged in this study were tagged within the boundaries of SOAR, an area where training exercises including the use of MFA sonar and a variety of other loud anthropogenic sound sources (e.g. explosions, acoustic counter-measures) occur regularly. The U.S. Navy Letter of Authorization suggests this could be as high as 2471 hours of ship-based MFA per year (an average of 6.8 hours/day), not including other sources such as active sonobuoys, dipping helicopter sonar, or submarine-based sonar [6]. Previous studies have suggested *Ziphius* may even alter their diving behavior in the presence of loud ships [21], which regularly pass through this region on their way to Los Angeles/Long Beach Harbor.

Most of our tagged whales predominately used SOAR, and the waters of the San Nicolas Basin which encompass it, during the time of tag transmission. In total, they were present within the San Nicolas Basin on 53% of days tags transmitted, and spent 71% of their time within the boundaries of the SOAR range when in the basin (Figure 1). While four whales left the Southern California Bight, two returned to SOAR during the transmission period. Zc016 was inside the San Nicolas Basin on 74% of days over the three months her tag was active. Previous and ongoing photo-identification related to this tagging effort have revealed the same whales on SOAR repeatedly across years, and at times *Ziphius* occur in higher densities on SOAR than have been reported anywhere else along the US West Coast – the region across which this population is managed [9], [22]. These findings suggest the San Nicolas Basin represents important habitat for these whales, despite its high level of acoustic disturbance. The reasons for this remain unclear, as the Southern California Bight is an area of complex oceanographic processes, and most productivity studies have occurred well above the depths at which these whales forage [23].

Given the acoustic sensitivity of beaked whales and other odontocetes, it is hard to imagine that sonar use does not displace whales at least occasionally, an effect that was suggested by the experimental exposures [3] and also by a satellite-tagged Blainville’s beaked whale that was exposed to MFA sonar in the Bahamas [24]. Though studies on longer-term, or larger scale displacement of disturbed cetaceans are limited, Morton and Symonds [25] demonstrated a significant decline in use of an area by killer whales while acoustic harassment devices were present, and harbor porpoises have been shown to abandon an area during loud pile driving operations [26]. Whatever displacement does occur in this population appears to be temporary. Unfortunately, with no data on beaked whale prey assemblages within the San Nicolas Basin, it is presently very difficult to quantify the effect this displacement might have on population health. All whales continued to forage even outside the San Nicolas Basin, so there is clearly suitable prey elsewhere.

Given the regular use of MFA sonar in the region [6], [7], tagged whales were almost certainly exposed at some point during data collection. Without a doubt, a valuable application of this data set would be a detailed assessment of whale behavior in the presence and absence of acoustic disturbance. Unfortunately, detailed, accurate records of sonar use across the entire spatial and temporal scales these tags were active are much less readily available than one might expect, particularly when the animals were outside of SOAR. Previous authors correlating beaked whale strandings with sonar records have encountered these limitations both in this region and elsewhere [4], [27]. Where available, records may include only the start time and location of MFA sonar use, despite activity that may last multiple hours, and during which ships may move large distances over variable courses. Single-ship events are often not captured. In light of this, an independent, comprehensive analysis of the entire dataset presented here in the context of sonar use is unlikely to ever be possible, especially given the apparent complexities of *Ziphius* response [3], which may be mediated by both source level and proximity, and would require detailed knowledge of vessel movements and sonar transmission schedules. Efforts to identify known sonar exposures in this dataset are underway, but they will involve only a subset of this dataset where major sources of acoustic disturbance - or just as importantly, lack thereof –can be accurately documented and independently verified.

Ultimately, the prolonged and recurrent use of the San Nicolas Basin by whales in this population suggests that *Ziphius* in this region have likely adapted to life with a certain amount of acoustic disturbance, and that local advantages may outweigh the costs it imposes. In light of this, the behavior of whales tagged in this region may not be representative of whales that are naive to such intense sounds. Given that whales tagged in this study far exceeded diving behavior previously described as extreme [2], the role humans might play in shaping this behavior can’t be discounted. Regardless, this long-term data set provides a much more complete picture of the true diving capabilities of *Ziphius* in a region of regular acoustic disturbance.
